# Optimal reference genes for RNA tissue analysis in small animal models of hemorrhagic fever viruses

**DOI:** 10.1038/s41598-023-45740-w

**Published:** 2023-11-08

**Authors:** Katherine A. Davies, Stephen R. Welch, Teresa E. Sorvillo, JoAnn D. Coleman-McCray, María Laura Martin, Julia M. Brignone, Joel M. Montgomery, Christina F. Spiropoulou, Jessica R. Spengler

**Affiliations:** 1 Viral Special Pathogens Branch, Division of High-Consequence Pathogens and Pathology, Centers for Disease Control and Prevention, Atlanta, GA USA; 2 U.S. Department of Agriculture, Agricultural Research Service, Zoonotic and Emerging Disease Research Unit, National Bio and Agro-Defense Facility, Manhattan, KS USA; 3 Departamento Investigación, Instituto Nacional de Enfermedades Virales Humanas (INEVH) “Dr. Julio I. Maiztegui”, Pergamino, Argentina

**Keywords:** Animal disease models, Viral pathogenesis

## Abstract

Reverse-transcription quantitative polymerase chain reaction assays are frequently used to evaluate gene expression in animal model studies. Data analyses depend on normalization using a suitable reference gene (RG) to minimize effects of variation due to sample collection, sample processing, or experimental set-up. Here, we investigated the suitability of nine potential RGs in laboratory animals commonly used to study viral hemorrhagic fever infection. Using tissues (liver, spleen, gonad [ovary or testis], kidney, heart, lung, eye, brain, and blood) collected from naïve animals and those infected with Crimean–Congo hemorrhagic fever (mice), Nipah (hamsters), or Lassa (guinea pigs) viruses, optimal species-specific RGs were identified based on five web-based algorithms to assess RG stability. Notably, the *Ppia* RG demonstrated stability across all rodent tissues tested. Optimal RG pairs that include *Ppia* were determined for each rodent species (*Ppia* and *Gusb* for mice; *Ppia* and *Hrpt* for hamsters; and *Ppia* and *Gapdh* for guinea pigs). These RG pair assays were multiplexed with viral targets to improve assay turnaround time and economize sample usage. Finally, a pan-rodent *Ppia* assay capable of detecting *Ppia* across multiple rodent species was developed and successfully used in ecological investigations of field-caught rodents, further supporting its pan-species utility.

## Introduction

Well-characterized animal models are critical translational research tools. They are widely used to examine disease pathogenesis, viral transmission kinetics, and to evaluate medical countermeasures. Studies involving animal models routinely use reverse transcription-quantitative polymerase chain reaction (RT-qPCR) assays to quantify RNA levels in samples, allowing elucidation of complex processes such as viral replication kinetics, host immune responses, and alterations to the cellular transcriptome. To accurately quantify and compare changes in tissue RNA levels both from an individual and between experimental subjects, it is vitally important to minimize inherent variations arising from sample collection and processing by normalizing data to a suitable standard reference gene (RG).

Historically, numerous genes have been utilized as RGs; common examples include *18S* RNA (*18S*), beta-actin (*Actb*), TATA-box binding protein (*Tbp*), glyceraldehyde-3-phosphate dehydrogenase (*Gapdh*), hypoxanthine–guanine phosphoribosyl transferase (*Hrpt*), and beta-2-microglobulin (*B2m*)^1,2^*.* However, many studies using these RGs do not demonstrate or reference appropriate validation data^1^. Furthermore, RG expression levels can vary depending on experimental factors such as cell type^3^; animal species^4^, age^5^, sex^6^, and diet^7^; and stressors such as drug treatment^8^ or infection^9^. It is therefore critically important to evaluate multiple RG candidates under a variety of conditions for their suitability as housekeeping genes for specific animal species.

**Figure 1:**
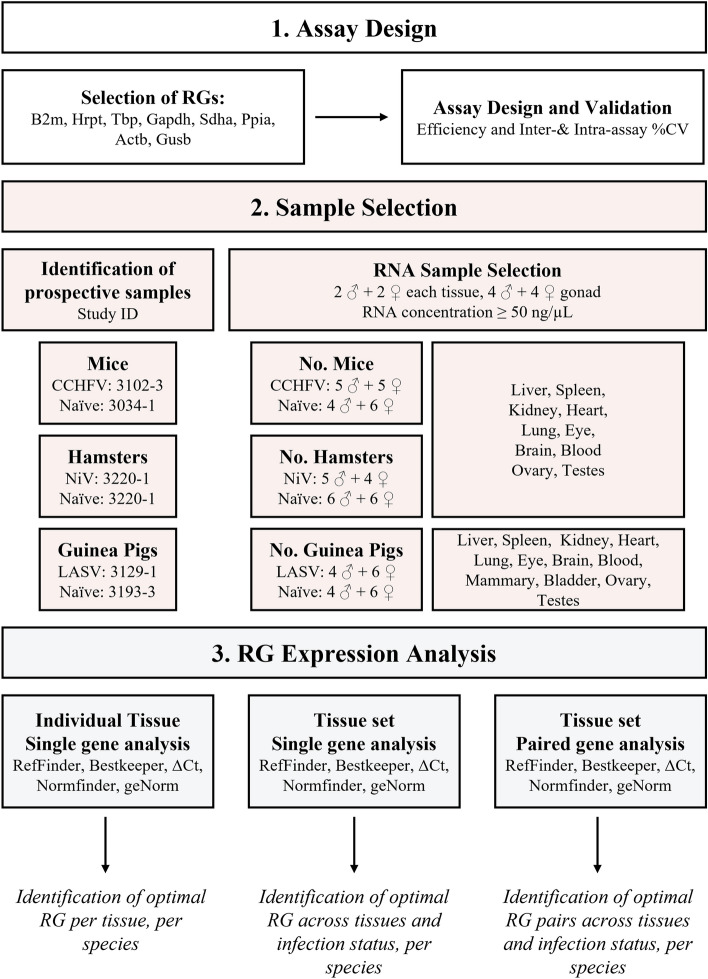
Sample selection and analysis flow chart. (1) Selection and design of reference gene (RG) assays against potential optimal RGs, followed by assay validation. (2) Selection of sample RNA for determining RG stability. Samples were selected from prospective studies with study IDs, number of male (♂) and female (♀) animals required, and target organs indicated. (3) RG analysis methods. First, RG stability is assessed in each individual tissue, then across all tissue sets; finally, paired gene analysis was carried out to find optimal RG pairs.

The ideal RG would be constitutively expressed and not be altered by disease state. During infection, viruses appropriate and modify host cellular and metabolic pathways to ensure efficient replication and to evade the immune system^10^. Therefore, it is essential to ensure that RG expression remains unchanged despite these modifications. Here, we investigated the suitability of nine potential RGs in three commonly used small animal models of viral hemorrhagic fever (VHF) to validate RGs for three highly pathogenic zoonoses: Crimean–Congo hemorrhagic fever virus (CCHFV) in mice^11^, Nipah virus (NiV) in hamsters^12^, and Lassa virus (LASV) in guinea pigs^13^. Multiple tissues (liver, spleen, gonad [ovary or testis], kidney, heart, lung, eye, brain, and blood) were evaluated both from naïve and infected animals to ensure the validity of the RG. To determine the optimal RG, five web-based algorithms—RefFinder, Bestkeeper, NormFinder, geNorm, and delta C_t_ (ΔC_t_) methods—were used to assess RG stability (Fig. 1).

## Methods

### Biosafety

All work with infectious virus or infected animals was conducted in a biosafety level 4 (BSL-4) laboratory at the Centers for Disease Control and Prevention (CDC) following established BSL-4 standard operating procedures approved by the Institutional Biosafety Committee. The CDC Institutional Biosafety Committee approved all recombinant virus work. All animal experiments were approved by the CDC Institutional Animal Care and Use Committee, performed in an AAALAC International-approved facility, and conducted in accordance with the *Guide for the Care and Use of Laboratory Animals*. The CDC is fully accredited by the AAALAC International.

### Origin of tissue RNA

The study aimed to use RNA from tissues harvested and archived during previous experimental studies and from tissues harvested from in-house colony animals humanely euthanized for study-independent health indications (e.g., advanced chronic disease). In this study, naïve animal tissue RNA was selected from animals that did not receive virus challenge. Infected animal tissue RNA samples were selected from animals that succumbed to lethal disease (NiV, CCHFV, or LASV) or displayed high viral RNA loads in tissues at endpoint (NiV) after infection with well characterized lethal challenge doses.

Infected mouse tissue RNA was obtained from experiments (Study# 3102-3) in which C57BL/6J mice (JAX stock No. 000664; male and female; 6 weeks old) transiently immunosuppressed with a type 1 interferon suppressing monoclonal antibody (5A3, Lienco)^14^ were infected intraperitoneally (IP) with 10^2^ TCID_50_ of recombinant CCHFV strain IbAr10200^15^ (CDC Virharv# 813730; GenBank KJ648914, KJ648915, and KJ648913), using 200 µL total volume per animal, divided bilaterally. Age-matched naïve mice (Study# 3034-1) were inoculated subcutaneously (SC) in the interscapular region with 100 µL of Dulbecco's Modified Eagle Medium (DMEM). At terminal or pre-determined endpoint, mice were euthanized by isoflurane inhalation followed by cervical dislocation.

Infected hamster tissue RNA was obtained from experiments (Study# 3220-1) in which HsdHan^®^:AURA Syrian hamsters (Envigo no. 8902F or 8902M; male and female; 5 weeks old) were intranasally (IN) infected with 10^6^ TCID_50_ of NiV strain Malaysia^16^ (CDC Virharv# 813744; GenBank AF212302), 100 µL total volume per animal, divided between the nares. Naïve hamsters (Study# 3220-1) were inoculated IN with an equivalent volume of DMEM. At terminal or pre-determined endpoint, hamsters were euthanized by isoflurane inhalation followed by confirmation of cardiac arrest.

Infected guinea pig tissue RNA was obtained from experiments (Study# 3129-1) in which strain 13/N guinea pigs (in-house CDC colony; male and female; 203–1195 days old) were inoculated SC in the interscapular region with 10^4^ TCID_50_ of recombinant LASV strain Josiah^17^ (CDC Virharv# 813752; GenBank HQ688673; HQ688675), 500 µL total volume per animal. Naïve animals (Study# 3193-3) were taken from the in-house colony (male and female; 879–1345 days old) and euthanized for humane reasons (e.g., age, injury, or underlying conditions). At terminal or pre-determined endpoint, guinea pigs were euthanized by isoflurane inhalation and intracardiac administration of sodium pentoparbital followed by confirmation of cardiac arrest.

### Animal husbandry

All animals were housed under climate-controlled conditions (range: 68–79 °C; 30–70% humidity) with a 12 h day/night cycle. All infected animals were housed in isolator caging systems containing a HEPA-filtered inlet and exhaust air supply. Both naïve and infected mice were group-housed (5–6 per cage) on corncob bedding (Bed-o’Cobs^®^ ¼”, Anderson Lab Bedding; Care Fresh, Healthy Pet; Enviro-Dry, Shepherd Specialty Papers) with cotton nestlets in an isolator caging system (Tecniplast GM500, West Chester, PA, USA), and provided sterilized, commercially available mouse chow and water ad libitum. Naïve and infected Syrian hamsters were group-housed (4–6 per cage) on corncob bedding, with wood blocks, seeds (given weekly), and plastic hide-aways, in an isolator caging system (Tecniplast GR900), and provided rodent chow (Teklad Rodent Diet 18% protein, Envigo) and water ad libitum*.* Naïve guinea pigs were housed in floor pens in BSL-2 segregated by sex, in a climate-controlled laboratory with a 12:12 h light cycle. Animals were provided with ad libitum feed (Guinea Pig Diet 5025) and water, timothy hay daily, and fresh vegetables 3 times a week. Infected guinea pigs were singly housed on soft pellets (paper chip; Carefresh), with Enviro-Dri wood blocks, in an isolator caging system (Tecniplast Blue Line 1500U), provided chow and water ad libitum as above, and both timothy hay and fresh produce daily. This study is reported in accordance with ARRIVE guidelines.

### PubMed query search for specifc reference genes

**Figure 2:**
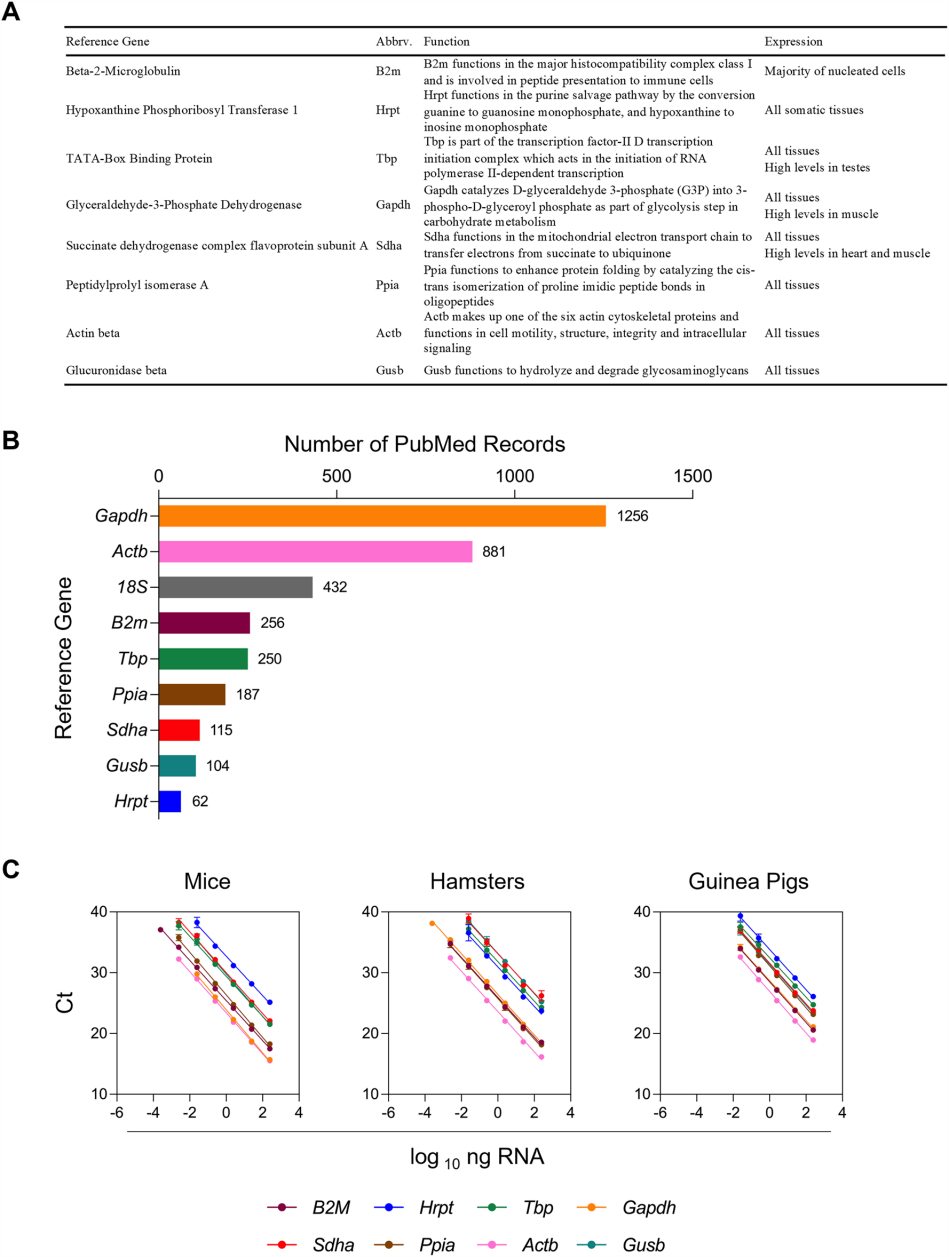
Reference gene targets chosen for gene stability investigation to use in RT-qPCR normalization. (**A**) Reference gene function and expression. Data were collated from the GeneCards Suite^18^ and the Mouse Genome Informatics Gene Expression Database^19^. (**B**) Number of results obtained from PubMed queries including the common names and abbreviations for each specifc RG (e.g., “18S” or “18SRNA” or “18S RNA”) and “housekeeping gene” or “reference gene.” (**C**) Standard curves were generated by making tenfold dilutions of 100 ng/µL total RNA isolated from the spleen of naïve mice, hamsters, or guinea pigs. Each point represents the mean of 8 independent RT-qPCR reactions, and error bars indicate the standard deviation from the mean.

To determine a relative level of how commonly RGs are used in literature, a PubMed search was conducted for each chosen RG. Queries included the common names and abbreviations of each specifc RG (e.g., “18S” or “18SRNA” or “18S RNA”) and “housekeeping gene” or “reference gene” (Fig. 2B).

### Assay design

Primer and probes were designed using Primer3 software^20^ against 8 target RGs (Fig. 2)—*B2m*, *Hrpt*, *Tbp*, *Gapdh*, *Sdha*, *Ppia*, *Actb*, and *Gusb*—using sequences specific for each species (Table 1). Pan-species (mouse, hamster, guinea pig) primer–probe sets targeting *Ppia* (Table 2) were designed using Primer3 software against a consensus sequence derived from the alignment of each species-specific mRNA sequence (Fig. S3). All primers and probes were synthesized by Integrated DNA Technologies.

**Table 1:** Reference gene assay primer sequences for each rodent species.

Oligo Name	Mouse (*Mus musculus*)		Hamster (*Mesocricetus auratus*)		Guinea pig (*Cavia porcellus*)	
	Primer sequence (5′-3′)	Conc. (µM)	Primer sequence (5′-3′)	Conc. (µM)	Primer sequence (5′-3′)	Conc. (µM)
*B2m_Fwd*	GCAAAGAGGCCTAATTGAAGTC	400	TATGTGTCGCAGTTCCATCC	400	CTATCTCCTGGTGCATGCTG	400
*B2m_Rev*	AGAAGTAGCCACAGGGTTGG	400	GACAGCTCCACTTTGTCCATC	400	ACTTGTTTGGGTCCCATTTG	400
*B2m_Prb*	TCACTGTGCCCAATGCTTAGCA	200	CCCACATTGAAATCGAGCTGCTG	200	TCACACATCACTCTGAGTGAACCCA	200
*Hrpt_Fwd*	CTGCCTCTGCCTCCTAAATG	400	AGCCTGTTGGGCTTACTTCC	550	CAGACTATCGTCGTCCTGCTC	400
*Hrpt_Rev*	GATTTAGGCCCAGTTCTTTCAC	400	ATCACGACGCTGGGACTG	400	GGTCATAGCCTGGTTCATCG	400
*Hrpt_Prb*	ATTAAAGGCGTGCGCCACCA	200	ACCGATTCCGTCATGGCGAC	450	TTCGGCTCGGTTATGGCGAC	200
*Tbp_Fwd*	CTGCTGTTGGTGATTGTTGG	400	AAGAGAGCCTCGGACAACTG	400	CATGGTGGTGGTGTGAGAAG	400
*Tbp_Rev*	AACTGGCTTGTGTGGGAAAG	400	TGCTGCTAACCTGGATTGTTC	400	TCTCAGTGCAGGAGAGTAGCC	400
*Tbp_Prb*	TGTTCAAGCCACCTGTACAATTGGA	200	TGGTGTGCACAGGAGCCAAGA	200	CAGCTCTTCTGGCCAGGCCC	200
*Gapdh_Fwd*	CATGGCCTCCAAGGAGTAAG	400	TGATGGGTGTGAACCATGAC	550	TGGCCTCCAAGGAGTAAGTG	400
*Gapdh_Rev*	TGTGAGGGAGATGCTCAGTG	400	CTAAGCAGTTGGTGGTGCAG	500	TGCAGTGAGGCCTAGTCTCC	400
*Gapdh_Prb*	AAACCCTGGACCACCCACCC	200	TCCCTCAAGATTGTCAGCAATGCA	350	CCAGCGAGCCCAGTGAGACC	200
*Sdha_Fwd*	GTCTCTGAGGGATTGGCTTG	400	TATGGGCGGACCTACTTCAG	400	GTGAGGGCGAGAGGTTTATG	400
*Sdha_Rev*	CAGTCCAGACTGATTTGACCAG	400	CTAAGTCCTGGCAAGGCAAG	400	CTTCCTTCACGGATCTCCAG	400
*Sdha_Prb*	AGCCCACTGTGGGCACCATC	450	ACAGCCATGGTCACCAGGGC	200	CTGGCATCCCGAGACGTCGT	200
*Ppia_Fwd*	ATGGCAAGACCAGCAAGAAG	400	ATCACCATTTCCGACTGTGG	400	GCCCTGTCATCTCTGCTCTC	400
*Ppia_Rev*	TCCTGAGCTACAGAAGGAATGG	400	ACAGAGGGAATGGTCTGGTG	400	TGCAATCCAGCTATGTTTGG	400
*Ppia_Rev*	CACCATTTCCGACTGTGGACAGC	200	TTCTTCTGACTCGACGGCCTATTACC	200	TGGGTTCCATATATCTTCCTTGTTCCC	200
*Actb_Fwd*	GATCATTGCTCCTCCTGAGC	400	AGCGCAAGTACTCTGTGTGG	400	TTCCAGCAGATGTGGATCAG	400
*Actb_Rev*	ACATCTGCTGGAAGGTGGAC	400	CATCGTACTCCTGCTTGCTG	400	AAGGGTGTAACGCAGCAAAG	400
*Actb_Prb*	TGGCTCCATCCTGGCCTCAC	200	TGGCTCCATCCTGGCCTCAC	200	TCGTGCACCGCAAATGCTTC	200
*Gusb_Fwd*	CCCAAGGGTTACTTTGTCCAG	400	CCTTGGAGGTGAGGATGATG	400	GCCCGCAGGCTACTACTTC	400
*Gusb_Rev*	TGGTATAGAGGACCACAGATCG	400	AGGAACTTGCTCTCGGTGAC	400	TTGGTCACAAAGGTCACTGG	400
*Gusb_Prb*	TTCTTCAACTATGCGGGACTGCA	200	ACCCTCCCTGTCGGGATTCG	200	AAGCCTTGGATCCCTCCCGG	200

Primer sequences for each reference gene of interest are indicated in the 5′–3′ direction. The final concentration (conc.) of each individual primer or probe per reaction is indicated in micromolar (μM). Primers were designed using Primer3 software against gene sequences available on Genbank; mice (*B2m*, NM_009735; *Hrpt*, AH003453; *Tbp*, NM_013684; *Gapdh*, GU214026; *Sdha*, NM_023281; *Ppia*, NM_008907; *Actb*, NM_007393; *Gusb*, NM_001357027), hamster (*B2m*, XM_005068531; *Hrpt*, XM_005085546; *Tbp*, NM_001281633; *Gapdh*, DQ403055; *Sdha*, DQ402977; *Ppia*, XM_005086775; *Actb*, NM_001281595; *Gusb*, XM_040731404), and guinea pig (*B2m*, NM_001172856; *Hrpt*, XM_003462671; *Tbp*, XM_013148964; *Gapdh*, NM_001172951; *Sdha*, DQ402978; *Ppia*, XM_003465805; *Actb*, NM_001172909; *Gusb*, XM_013148254).

**Table 2:** Pan-rodent *Ppia* and viral target multiplex assay primer sequences.

Multiplex assay primer sequences		
Oligo name	Primer sequence (5′-3′)	Conc. (µM)
*Pan_Ppia_Fwd*	CCCACCGTGTTCTTCGAC	400
*Pan_Ppia_Rev*	TCCTTTCTCTCCAGTGCTCAG	400
*Pan_Ppia_Prb*	GAGCCCTTGGGCCGCGTCTC	200
*CCHFV_Fwd*	CAGGACATGGACATAGTGGC	400
*CCHFV_Rev*	ATTGCCCTTGACGTTGTAGG	400
*CCHFV_Prb*	CCCTTGTTGGCAAGCAATCCC	200
*NiV_Fwd*	CTGGTCTCTGCAGTTATCACCATCGA	400
*NiV_Rev*	ACGTACTTAGCCCATCTTCTAGTTTCA	400
*NiV_Prb*	CAGCTCCCGACACTGCCGAGGAT	200
*LASV_Fwd*	GGAAGCCACAGAAAGCTGAC	400
*LASV_Rev*	GGAGTGCATCAATGACAGCA	400
*LASV_Prb*	AAATCCCTGCAGTCGGCAGG	200

GenBank accession numbers used to produce a consensus sequence for designing primers and probes against *Ppia* from multiple rodent species were NM_008907 (mouse), XM_005086775 (hamster), and XM_003465805 (guinea pig). Primer sequences are indicated in the 5′–3′ direction. The final concentration (conc.) of each individual primer or probe per reaction is indicated in micromolar (μM).

### RNA extraction

RNA was extracted from either homogenized tissue (~ 1 mm^3^ sections) or 50 μL of whole blood in lithium heparin (mice and hamsters), or from 50 to 125 μL whole blood in EDTA (guinea pigs), using the MagMax-96 Total RNA isolation kit (Thermo Fisher Scientific) on a 96-well ABI MagMax extraction platform. Samples were treated with DNase (Lucigen) and eluted in 75 μL MagMax elution buffer. RNA was quantified using the Nanodrop 8000 spectrometer (Thermo Fisher Scientific). RNA purity was assessed and was found to have an average absorbance of 1.99 (A_260_/A_280_) with a standard deviation of 0.17. Extracted RNA was kept at − 80 °C for long-term storage.

### RT-qPCR

The equivalent of 100 ng RNA from each tissue type selected was analyzed by RT-qPCR to assess RG stability. These RT-qPCRs were performed using the OT-2 liquid handling platform (Opentrons) to reduce pipetting and volume errors. Assays were carried out using either in-house desgined primer sets (Tables 1 and 2) or a commercially available Eukaryotic 18S rRNA Endogenous Control (Thermo Fisher). RT-qPCRs were run on the CFX384 or CFX96 Real-Time PCR Detection systems (BioRad) with 10 μL reaction volume using the SuperScript III Platinum One-Step qRT-PCR kit (Thermo Fisher). Each PCR reaction was performed in duplicate. Thermal cycling conditions were as follows: 50 °C reverse transcription step for 15 min, 95 °C denaturation for 2 min, followed by 40 cycles of 95 °C for 15 s and 60 °C for 30 s. Primer–probe set sequences and concentrations can be found in Tables 1 and 2.

### Assay validation

Singleplex assay validation was performed on RNA harvested from the spleen of naïve hamsters, mice, or guinea pigs diluted serially tenfold from 100 ng/μL. In addition, singleplex and multiplex assay validations were performed on RNA harvested from the spleens of animals infected with CCHFV (mice), NiV (hamsters), or LASV (guinea pigs) diluted serially tenfold from 100 ng/μL. Efficiency calculations and inter- and intra-assay variability were determined from sample standard curves generated from at least five technical replicates, utilizing a minimum of three thermocyclers and three operators to account for both human and machine-derived variability. RT-qPCR efficiency was calculated from the mean of each dilution series, and the inter- and intra-assay variability were assessed using percentage correlation of variance (% CV).

### Variability analysis

C_t_ values were determined using the Bio-Rad CFX Manager 3.1 software by setting a standard baseline threshold level for each primer probe set. Analysis was carried out on mean C_t_ values of duplicate reactions. The stability of each RG was assessed using the web-based RefFinder tool (http://blooge.cn/RefFinder/?type=reference)^21,22^. This web-based platform also incorporates the following methods: comparative ΔC_t_ method^23^, Bestkeeper^24^, NormFinder^25^, and geNorm^26^. Analyses were initially carried out using data sets from each individual tissue, followed by analysis across all tissue types. Analyses were carried out on naïve animals and infected animals separately, before combining for further evaluation.

### RNA isolation and RT-qPCR from wild rodents

Field-caught rodents were identified by morphological characteristics or through molecular amplification of mitochondrial DNA cytochrome B gene. RNA was extracted from 100 µL rodent blood using TRI Reagent (Sigma-Aldrich) according to the manufacturer’s instructions. RT-qPCRs were performed using the Luna Probe One-Step RT-qPCR kit (New England BioLabs) with 20 μL reaction volume containing 5 µL extracted RNA. Thermal cycling conditions were as follows: 55 °C reverse transcription step for 10 min, 95 °C denaturation for 1 min, followed by 45 cycles of 95 °C for 10 s and 60 °C for 30 s. Samples were considered positive if values obtained were less than 35 C_t_.

### Statistical analyses

Data distribution was assessed using the Anderson–Darling normality test and confirmed to have normal distribution using a significance level of α = 0.05. Statistical analyses were carried out in GraphPad Prism v9.4.1.

## Results

### Evaluation of eight species-specific reference gene assays

Commercial assays are available for select commonly used RGs, such as *18S*. However, the availably of commercial assays for other candidate RGs is limited, especially for use across multiple rodent species. Here, we developed species-specific assays against 8 candidate RGs that are commonly used in the literature^1,23^ (*B2m*, *Tbp*, *Gapdh*, *Actb*, *Hrpt*, *Sdha*, *Ppia*, and *Gusb*) (Fig. 2B). Efficacy and variance of these assays were validated by analyzing standard curves generated by tenfold serial dilution of total RNA harvested from the spleen of naïve animals (Figs. 1-1 and 2C). Spleen RNA from naïve (uninfected) animals was chosen as the test material due to the consistently high concentrations of total RNA isolated from this tissue across all species. All species-specific singleplex assays were calculated to have efficiencies between 90 and 105% and the intra- and inter-assay variances were calculated to be < 3% CV and < 2% CV for all assays, respectively. Assay parameters are shown in Table 3. These in-house species-specific assays have now been evaluated to accurately amplify targets and therefore are appropriate to use to determine relative expression levels of RGs in tissues.

**Table 3:** Reference gene assay primer efficiency and variability.

	Singleplex			
	Efficiency (%)	R-squared	Intra-assay variability (%)	Inter-assay variability (%)
Mouse (*Mus musculus*)				
*B2m*	101.81	0.9971	1.47	0.78
*Hrpt*	103.21	0.9928	0.60	1.55
*Tbp*	103.26	0.9984	0.42	1.16
*Gapdh*	103.41	0.9987	0.55	0.85
*Sdha*	103.48	0.9818	0.39	1.91
*Ppia*	92.97	0.9986	0.51	1.08
*Actb*	100.45	0.9973	0.47	1.02
*Gusb*	93.63	0.9983	0.31	1.12
Hamster (*Mesocricetus auratus*)				
*B2m*	98.89	0.9999	2.64	0.92
*Hrpt*	103.21	0.9928	0.60	1.55
*Tbp*	101.23	0.9990	0.26	0.94
*Gapdh*	91.34	0.9982	0.42	1.18
*Sdha*	36.95	0.9917	0.36	1.40
*Ppia*	99.72	0.9982	0.34	1.56
*Actb*	97.56	0.9993	0.52	0.87
*Gusb*	93.63	0.9983	0.31	1.12
Guinea pig (*Cavia porcellus*)				
*B2m*	99.84	0.9998	0.92	0.89
*Hrpt*	99.11	0.9997	0.53	1.29
*Tbp*	91.10	0.9939	0.55	1.37
*Gapdh*	99.93	0.9992	0.57	1.65
*Sdha*	102.60	0.9992	0.27	1.14
*Ppia*	99.61	0.9992	0.50	1.08
*Actb*	97.52	0.9997	0.31	1.07
*Gusb*	98.52	0.9991	0.50	1.32

Assay parameters were determined from standard curves generated by tenfold dilutions of 100 ng/µL total RNA isolated from spleens of naïve mice, hamsters, or guinea pigs. Intra- and inter-assay variability was assessed using percentage correlation of variance (% CV).

### Determining relative expression levels of candidate reference genes for use on tissue RNA extracted from naïve or VHF-infected rodents

Once the candidate RGs were validated, we then determined expression of these 8 in-house species-specific RG assays and a pan-species commercial assay targeting *18S* RNA in RNA isolated from liver, spleen, ovary, testis, kidney, heart, lung, eye, and brain (all species), plus mammary gland and bladder (guinea pigs). Furthermore, to ensure that disease did not alter RG expression, gene levels were evaluated in tissues from naïve (uninfected), infected, or both naïve and infected animals for each virus/rodent model pair: CCHFV-infected mice, NiV-infected hamsters, and LASV-infected guinea pigs. RG expression was analyzed by RT-qPCR using 100 ng RNA harvested from tissue. Sample size for each virus/rodent model pair and infection status was n = 4 (2 male and 2 female) for all tissues except gonad; for gonad tissue, n = 8 (4 males and 4 females).

**Figure 3:**
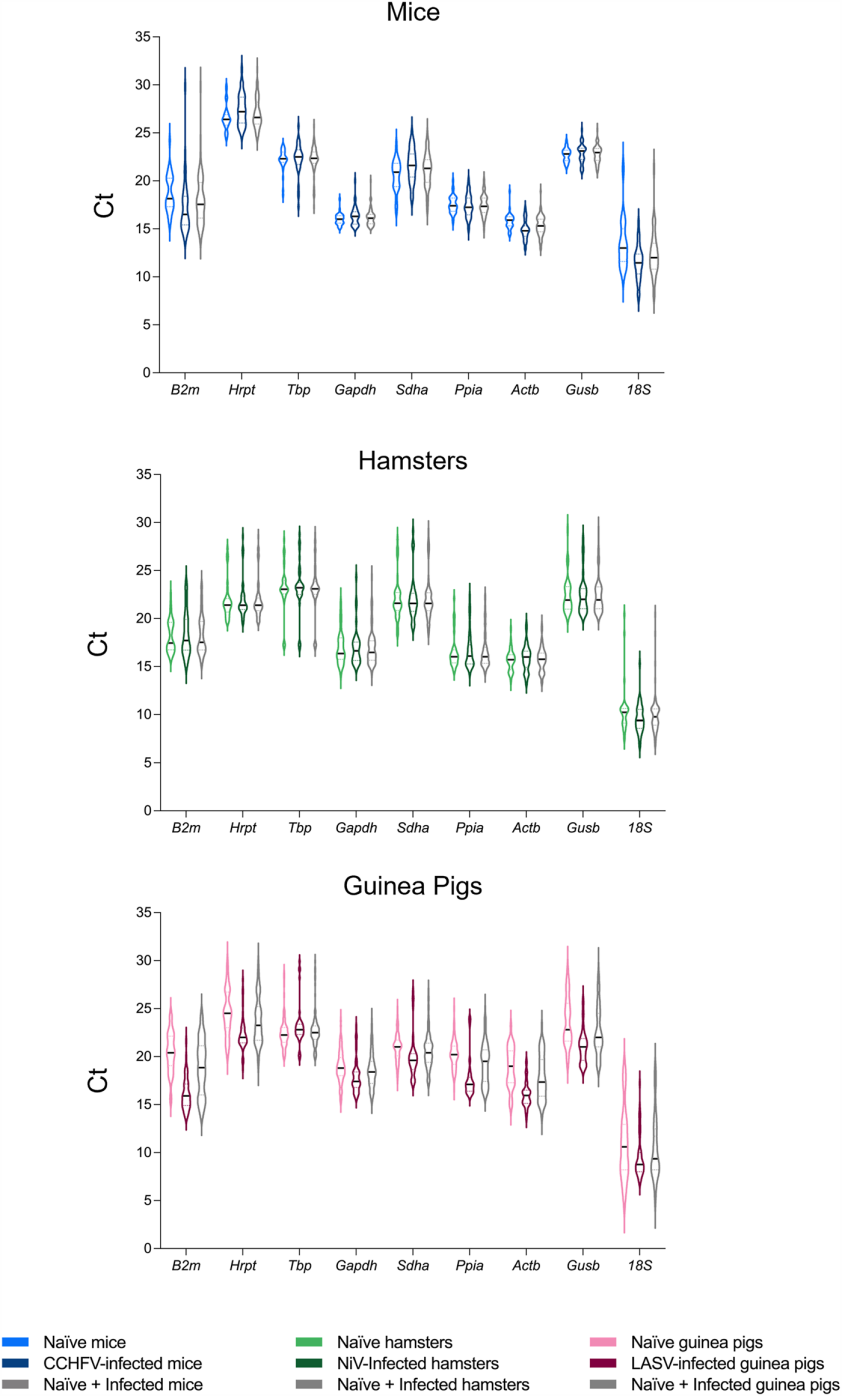
Variation of reference gene expression across all tissues isolated from hamsters, mice, and guinea pigs. Expression profiles of 8 RGs in 100 ng of total RNA across all tissues collected from either naïve animals; animals infected with NiV-M (hamsters), CCHFV (mice), or LASV (guinea pigs); or naïve + infected combined for each of the virus/rodent models. Cycle threshold (C_t_) values (n = 36–132) are presented as extended violin plots with median values indicated by a line.

Analyses revealed a broad range of C_t_ values across the nine RGs analyzed. The lowest C_t_ values, inclusive of naïve and infected tissue RNA, were given by the *18S* assay, with values ranging 7.8–21.7 (mouse), 6.8–20.4 (hamster), and 4.4–19.1 (guinea pig). These values also represent the largest range in C_t_ values obtained for an individual RG across all tissues, indicating high levels of variability in RG expression, making these less suitable for data normalization. Suboptimal large ranges in C_t_ values were also observed with *B2m* in mice (13.9–29.8), *Tbp* in hamsters (16.9–28.8), and *Hrpt* in guinea pigs (18.7–30.1). More ideal C_t_ ranges (i.e., those with a narrower range) were identified for the following RGs: *Gusb* (21.0–25.3), *Gapdh* (15.1–20.0) and *Ppia* (14.9–20.1) in mice; *Actb* (13.4–19.4) and *Ppia* (14.3–22.3) in hamsters; and *Gapdh* (15.3–23.8) and *Ppia* (15.8–25.0) in guinea pigs (Fig. 3, Fig. S1, Table S1).

### Comparative stability of reference genes across tissue types in naïve and VHF-infected mice, hamsters, and guinea pigs

To determine the stability of RGs, we applied 5 commonly used analysis methods available through a web-based platform: RefFinder analysis^21,22^, the comparative ΔC_t_ method^23^, Bestkeeper analysis^24^, Normfinder analysis^25^, and geNorm analysis^26^. These methods are readily utilized across studies of RG analysis and are freely available. These analyses provide a statistical basis to measure the variability of each gene’s expression and output this as a value of stability. Here, the term stability will be used to describe this variability. A stable gene would be given a low score, indicating that it has minimal variability across the tissues and experimental conditions tested. RefFinder analysis uses a combination method in which the scores determined from each of the other analysis methods are weighted and the stability value is calculated from the geometric mean. Comparative ΔC_t_ compares C_t_ values and determines the relative expression of pairs of genes within the sample. Bestkeeper analyzes gene stability through the standard deviation of sample C_t_ values. Normfinder provides a stability value (S), determined as the lowest variation between the inter- and intra-group variations. GeNorm analysis ranks gene stability based on the pairwise variation between two genes. For each method, a lower score indicates a more stable expression, and higher scores indicate less stable expression. For Bestkeeper, Normfinder, and geNorm, genes with acceptable stability score below 1.

Due to the extensive range of tissues routinely examined in viral pathogenesis and medical countermeasure studies, we aimed to determine the most appropriate RG to implement across multiple tissue types in naïve and virus-infected animals for each of the previously investigated virus/rodent model pairs. Prior to assessing all tissues collectively, we first assessed the stability of each RG expression by tissue type to ensure that there were no tissue-specific outliers. We found that, occasionally, specific tissues in specific animal species (e.g., blood from mice and lungs from guinea pigs) had more variation in RG expression, but overall in individual tissues, irrespective of species or infection status, most RGs had acceptable stability scores (Fig. S2). Exceptions included *B2m*, which often ranked low amongst the tested RGs (particularly in mouse tissues), and *18S*, which consistently demonstrated unstable expression scores across multiple tissue types and species using the 5 analysis methods. For *18S*, this finding, and the previously noted wide range of C_t_ values (Fig. S2, Table S1), indicated extensive variation and instability in gene expression; thus, *18S* was removed from further analysis.

**Figure 4:**
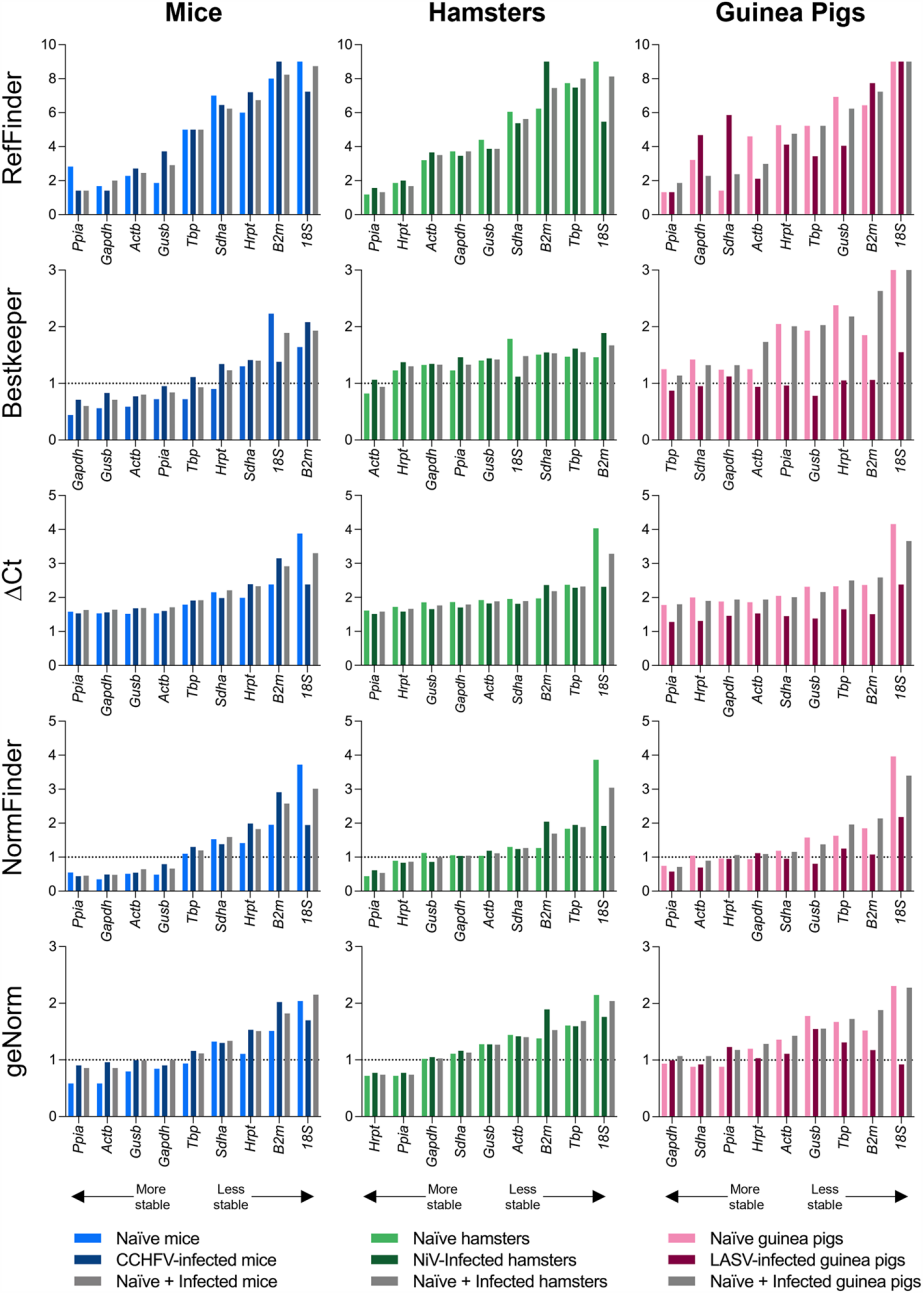
Stability of reference genes in RNA harvested from hamster, mouse, and guinea pig tissues. Stability of RGs in 100 ng total RNA across multiple tissue types from mice (total replicates per gene: n = 76), hamsters (total replicates per gene: n = 80), and guinea pigs (total replicates per gene: n = 94) was assessed using the web-based algorithms RefFinder, Bestkeeper, ΔC_t_, Normfinder, and geNorm. Lower gene stability values indicate higher gene stability. Dashed line indicates the greatest acceptable stability value, where appropriate.

After ensuring consistency across tissue types, we carried out stability analyses utilizing C_t_ values obtained collectively across all tissue RNAs from naïve and infected animals for each of the virus/rodent model pairs. Using this approach, we determined that the RGs demonstrating highest levels of stability across all tissues were: *Ppia* (determined by RefFinder, ΔC_t_, Normfinder, and geNorm) and *Gapdh* (Bestkeeper) in mice; *Ppia* (RefFinder, ΔC_t_, and Normfinder), *Actb* (Bestkeeper), and *Hrpt* (geNorm) in hamsters; and *Ppia* (RefFinder, ΔC_t_, and Normfinder), *Tbp* (Bestkeeper), and *Gapdh* (geNorm) in guinea pigs (Fig. 4). *Ppia* was the most stable RG when analyzed across multiple tissues and infection conditions among the rodent species examined here. We also note that other RGs demonstrating satisfactory stability scores could be used as acceptable alternates to *Ppia* (Fig. 4).

### Optimal pairing of reference genes for tissue RNA analysis of naïve and VHF-infected rodents

**Figure 5:**
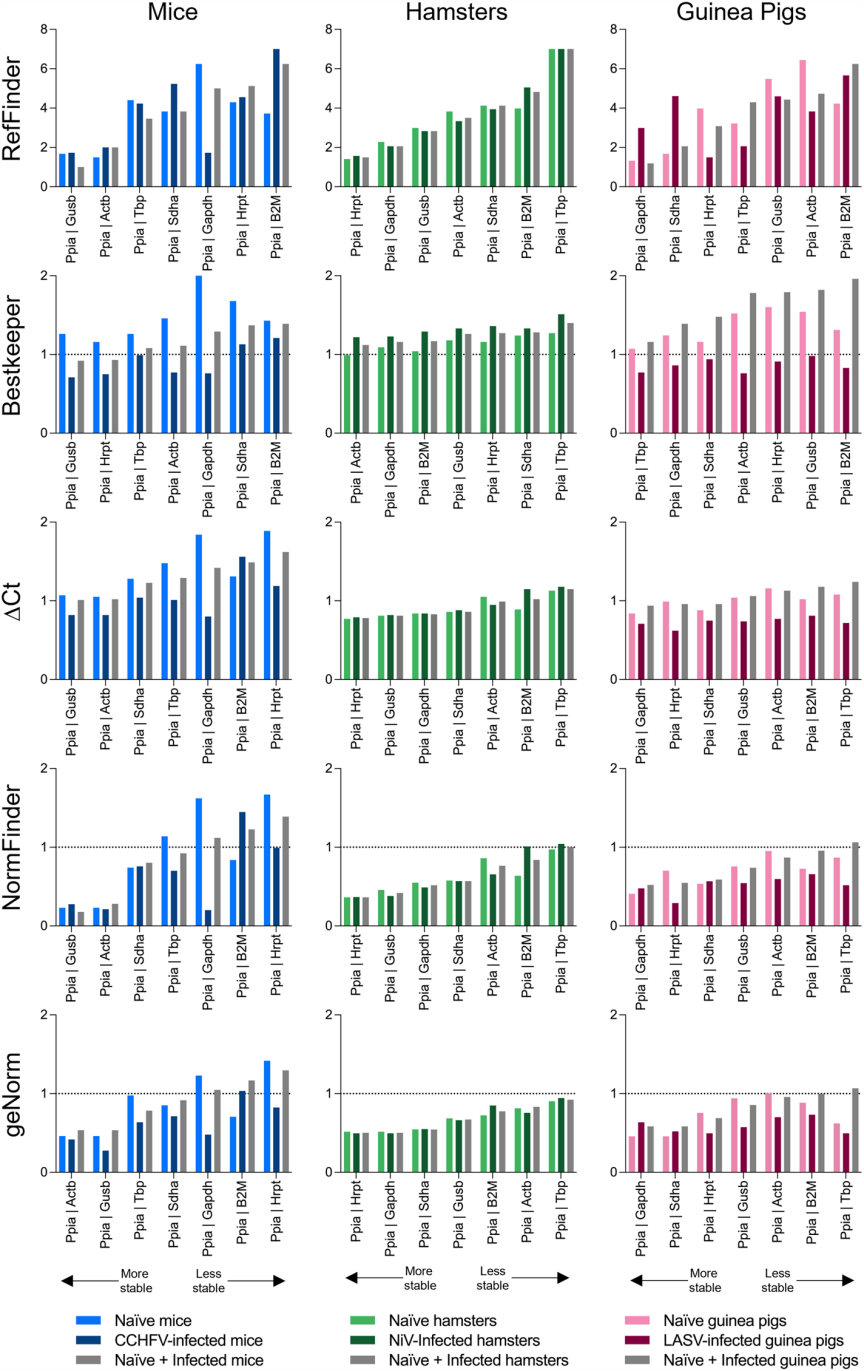
Stability of reference gene pairs in RNA harvested from hamster, mouse, and guinea pig tissues. Stability of RG pairs in 100 ng total RNA across multiple tissue types from mice (total replicates per gene: n = 76), hamsters (total replicates per gene: n = 80), and guinea pigs (total replicates per gene: n = 94) was assessed using the web-based algorithms RefFinder, Bestkeeper, ΔC_t_, Normfinder and geNorm. Lower gene stability values indicate higher gene stability. Dashed line indicates the greatest acceptable stability value, where appropriate.

We identified several RGs that may be appropriate for normalizing RT-qPCR data in these infection models when used alone; however, the use of multiple RGs is recommended to provide greater accuracy for RT-qPCR normalization than using a single RG^2,26^. Therefore, we identified RGs pairs using the same web-based analysis software as described above utilizing C_t_ values obtained from the analyses of tissue RNA from naïve and infected animals for each virus/rodent model pair. As *Ppia* was determined to be the most suitable RG candidate across multiple species, we focused on RG pairs that included *Ppia* to provide a level of continuity across the different rodent models used in our studies (Fig. 5).

In mice, the gene pair *Ppia* and *Gusb* (naïve + infected scores: RefFinder, 1.0; Bestkeeper, 0.92; ∆C_t_, 1.01; Normfinder, 0.18; geNorm, 0.54) demonstrated optimal stability, with *Ppia* and *Actb* (naïve + infected scores: RefFinder, 2.0; Bestkeeper, 0.93; ∆C_t_, 1.02; Normfinder, 0.28; geNorm, 0.54) also demonstrating high levels of stability (Fig. 5, left column). For hamsters several gene pairs were determined as suitable for use as RGs, including *Ppia* and *Actb* (naïve + infected scores: RefFinder, 3.5; Bestkeeper, 1.12; ∆C_t_, 0.99; Normfinder, 0.76; geNorm, 0.83), *Ppia* and *Gusb* (naïve + infected scores: RefFinder, 2.8; Bestkeeper, 1.26; ∆C_t_, 0.81; Normfinder, 0.42; geNorm, 0.67), and *Ppia* and *Gapdh* (naïve + infected scores: RefFinder, 2.1; Bestkeeper, 1.16; ∆C_t_, 0.83; Normfinder, 0.52; geNorm, 0.50)*,* with the highest scoring gene pair being *Ppia* and *Hrpt* (naïve + infected scores: RefFinder, 1.5; Bestkeeper, 1.27; ∆C_t_, 0.78; Normfinder, 0.36; geNorm, 0.50) (Fig. 5, center column). For guinea pigs, *Ppia* and *Gapdh* (naïve + infected scores: RefFinder, 1.2; Bestkeeper, 1.39; ∆C_t_, 0.94; Normfinder, 0.52; geNorm, 0.58) demonstrated high levels of stability, though other gene pairs may also be suitable (Fig. 5, right column).

### Validation of triplex assays for the detection of viral target and reference genes in VHF-infected rodent tissue samples

**Figure 6:**
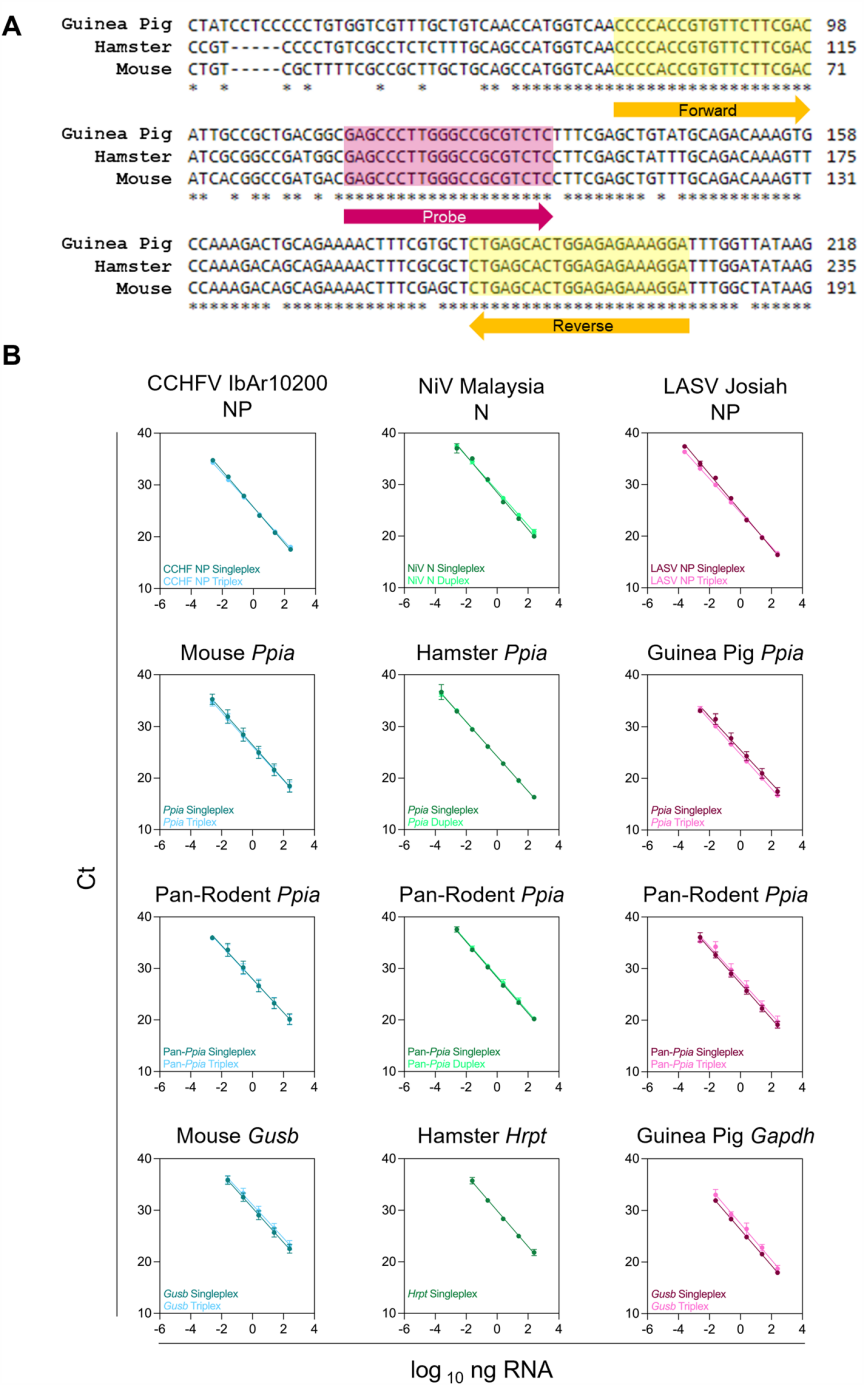
Viral assays can be multiplexed with RG assays. (**A**) Multiple sequence alignment of *Ppia* coding sequences from mice (Genbank #NM_008907), hamsters (Genbank #XM_005086775), and guinea pigs (Genbank #XM_003465805) using ClustalW^27^. Asterisk (*) denotes conserved sequences. Locations of pan-species *Ppia* primers and probe are indicated. (**B**) Standard curves generated by tenfold dilutions of 100 ng/µL total RNA isolated from the spleen of CCHFV-infected mice, NiV-infected hamsters, or LASV-infected guinea pigs. Each assay was run as singleplex or was multiplexed (CCHFV and LASV: viral targets with species-specific RG and pan-rodent *Ppia* or species specific *Ppia*; NiV: viral target with pan-rodent *Ppia* or species-specific *Ppia*). Standard curves from both singleplex and multiplexed assays are shown. Each point represents the mean of 5–12 independent RT-qPCR reactions.

As animal studies can generate vast numbers of samples requiring analysis, we aimed to multiplex the optimal paired RG assays identified here with specific VHF viral targets to allow rapid sample processing and conservation of material. A pan-rodent *Ppia* assay was designed to detect *Ppia* across mouse, hamster, and guinea pig tissues, targeting a conserved region of this gene (Fig. 6A). The pan-*Ppia* assay demonstrated 90–105% efficiency across these tissues, with intra- and inter-assay variations of < 5% CV (Table 4). *Ppia* assays (species-specifc or pan-rodent) were combined with our previously determined optimal species RG assays and with assays targeting CCHFV, NiV, and LASV.

**Table 4:** Multiplex reference gene assay primer efficiency and variability.

	Infected tissue singleplex					Infected tissue multiplex			
	Efficiency (%)	R-squared	Intra-assay variability (%)	Inter-assay variability (%)		Efficiency (%)	R-squared	Intra-assay variability (%)	Inter-assay variability (%)
*Mouse (Mus musculus)*					*Mouse (Mus musculus)—Triplex*				
*CCHFV*	93.3	0.9991	0.26	0.55	*CCHFV*	101.5	0.9996	0.52	0.75
*Gusb*	99.1	0.9997	0.46	2.99	*Gusb*	103.9	0.9982	0.94	3.09
*Ppia*	97.8	0.9997	0.42	4.65	*Ppia*	101.0	0.9998	0.37	3.66
*Pan.Ppia*	103.0	0.9972	0.25	3.73	*Pan.Ppia*	104.5	0.9980	2.00	3.63
*Hamster (Mesocricetus auratus)*					*Hamster (Mesocricetus auratus)—Duplex*				
*NiV*	90.7	0.9927	0.52	2.90	*NiV*	98.31	0.9994	0.84	1.79
*Hrpt*	94.0	0.9986	0.55	1.60	*Hrpt*	N/A	N/A	N/A	N/A
*Ppia*	98.1	0.9995	0.62	1.17	*Ppia*	98.80	0.9998	0.30	0.85
*Pan.Ppia*	94.3	0.9989	0.54	1.33	*Pan.Ppia*	95.30	0.9998	0.99	1.81
*Guinea pig (Cavia porcellus)*					*Guinea pig (Cavia porcellus)—Triplex*				
*LASV*	90.6	0.9976	0.31	0.83	*LASV*	101.1	0.9999	0.76	1.87
*Gapdh*	94.3	0.9998	0.38	1.31	*Gapdh*	92.9	0.9967	2.33	3.08
*Ppia*	104.3	0.9917	0.41	3.44	*Ppia*	99.5	0.9999	0.29	1.28
*Pan.Ppia*	96.3	0.9996	0.5	2.50	*Pan.Ppia*	100.9	0.9865	4.47	4.18

Assay parameters were determined from standard curves generated by tenfold dilutions of 100 ng/µL total RNA isolated from spleens of CCHFV-infected mice, NiV-infected hamsters, or LASV-infected guinea pigs. Each assay was run as singleplex or was multiplexed (CCHFV and LASV: viral targets with species-specific RG and pan-rodent *Ppia* or species-specific *Ppia*; NiV: viral target with pan-rodent *Ppia* or species-specific *Ppia*). Intra- and inter-assay variability was assessed for both singleplex and multiplex assays using % CV.

To detect CCHFV in mouse samples, the CCHFV nucleocapsid protein (NP) gene assay was combined with mouse *Gusb* and mouse *Ppia* or pan-rodent *Ppia.* To detect LASV in guinea pig samples, the LASV NP gene assay was combined with guinea pig *Gapdh* and guinea pig *Ppia* or pan-rodent *Ppia.* To detect NiV in hamster samples, the NiV nucleoprotein (N) gene assay was only combined with with hamster *Ppia* or pan-rodent *Ppia*, as attempts to multiplex with hamster *Hrpt* were unsuccessful due to primer-primer interactions. These multiplexed assays were validated by RT-qPCR on tenfold serially diluted RNA isolated from tissues of infected animals to generate a standard curve. Concurrently, singleplex assays were carried out for each target of interest to ensure that C_t_ values differed < 1 C_t_ between singleplex and multiplex assays (Fig. 6B). All single- and multiplexed assays were calculated to have efficiencies of 90–105%, and the intra- and inter-assay variations were calculated to be < 5% CV. Assay parameters can be seen in Table 4.

### Pan-rodent *Ppia* assay can be used to detect rodent RNA in ecological field studies

To further the utility of the pan-rodent *Ppia* assay, we generated a sequence logo^28^ of 26 rodents *Ppia* gene sequences from data available from Genbank (Table 2, Fig. S4A). The pan-rodent *Ppia* assay primers demonstrated a high level of complementarity to these rodent sequences. Therefore, we theorized they could be used as positive control RGs during rodent sampling in the field, for example, during epidemiological studies of rodent-borne viruses. In a retrospective study of rodent viral reservoirs in Argentina, 31 blood specimens were analyzed representing the different rodent genotypes and their distribution in the country. *Ppia*-positive RT-qPCR results were obtained for *Calomys boliviae*, *Holochilus chacarius*, *Necromys lasiurus*, *Oligoryzomys* sp., (including *O. nigripes*, *O. chacoensis*, *O. flavescens*, and *O. longicaudatus*), and *Oxymycterus rufus*. The only rodent species that was negative on the pan-rodent *Ppia* assay was *Akodon azarae*, although other *Akodon* sp. (only identified to genus level) demonstrated positive results (Fig. S4B). These results suggest that the pan-rodent *Ppia* assay is able to detect *Ppia* across a wide range of rodents and may have further utility outside of the research laboratory.

## Discussion

In vivo data regarding RG stability have only been described for a limited number of animal models of infectious disease thus far, including murine cytomegalovirus in the mouse model^9^; Marek’s disease virus infection in chickens^29^; influenza in mice (with kidney yang deficiency syndrome)^30^; and vesicular stomatitis virus in Syrian golden hamsters^31^. The impact of VHF infection on RG expression in animal models has not yet been reported, likely due to limited facilities and the time-consuming nature of conducting in vivo experiments in the high-containment environment. We aimed to utilize historical archived tissue RNA from CCHFV, NiV and LASV studies to identify RGs suitable for normalization when performing RT-qPCR analyses of RNA samples harvested from small animal models of VHF. Five web-based platforms (RefFinder, Bestkeeper, ∆C_t_, Normfinder, and geNorm) were used to analyze the stability of eight RGs (*B2m*, *Hrpt*, *Tbp*, *Gapdh*, *Sdha*, *Ppia*, *Actb*, and *Gusb*) across multiple tissue types of mice, hamsters, and guinea pigs.

Viral infections are known to modify the expression of host genes, including those often used as RGs. In lungs of influenza A-infected mice, expression of *Gapdh* and *Actb* becomes less stable than in lungs from naïve animals^30^; we did not observe this destabilization during CCHFV infection. Additionally, assessment of RGs in naïve and VSV-infected Syrian hamster characterized *B2m* as more stable and *Hrpt* as less stable^31^, whereas we found *Hrpt* to be more stable and *B2m* less stable in naïve and NiV-infected Syrian hamsters. These differences underscore the importance of assessing RG stability under the appropriate experimental conditions due to differential modifications of host gene expression induced by different viruses.

Our work highlights how choice of RG alone may introduce discrepancy in data. Advances in animal model development are often hampered by experimental design-associated variability in outcome and virological indices. We found that commonly used RGs, including *18S* and *B2m,* may be unsuitable for use in rodent models and potentially other species as well, as they exhibited large variations within and across tissues. This variability could result in further challenges when comparing data within or between experimental groups or between studies in which these RGs were used. In contrast, we established that the RG *Ppia* was stably expressed across all tested samples from naïve and infected mice, hamsters, and guinea pigs and serves as a suitable choice for use in these species. *Ppia*, though not as commonly used an RG as *18S*, *B2m*, *Gapdh*, *Actb*, or *Tbp*^1^, has also been previously identified as a stably expressed gene in many small animal models, including mice^7,32–34^ and rats^35^, and has been posited as a preferred RG in the analysis of human tissue^36,37^. Often a single RG is used in tissue analyses. To further increase confidence in samples analyzed with the use of *Ppia*, we determined optimal gene pairs to use as RGs for each species: *Ppia* and *Gusb* for mice, *Ppia* and *Hrpt* for hamsters, and *Ppia* and *Gapdh* for guinea pigs.

To our knowledge, this is the first report to investigate stable expression of RGs to identify appropriate controls for normalizing RT-qPCR data in studies of VHF infection in animal models. Furthermore, we demonstrate the validation of RG and viral target multiplex assays in each model species, providing a strong framework for further research into VHF pathogenesis and therapeutics evaluation. Finally, we describe development and use of *Ppia* as a pan-rodent assay for wide application in both laboratory animals and wild rodent species.

### Supplementary Information

Supplementary Figures.

Supplementary Table S1.

## Data Availability

The datasets used and/or analysed during the current study are available from the corresponding author on reasonable request.

## References

[1] Huggett J, Dheda K, Bustin S, Zumla A (2005). Real-time RT-PCR normalisation; strategies and considerations. Genes Immun..

[2] Bustin SA (2013). The need for transparency and good practices in the qPCR literature. Nat. Methods.

[3] Rácz GA, Nagy N, Tóvári J, Apáti Á, Vértessy BG (2021). Identification of new reference genes with stable expression patterns for gene expression studies using human cancer and normal cell lines. Sci. Rep..

[4] Hruz T (2011). RefGenes: Identification of reliable and condition specific reference genes for RT-qPCR data normalization. BMC Genomics.

[5] Uddin MJ (2011). Age-related changes in relative expression stability of commonly used housekeeping genes in selected porcine tissues. BMC Res. Notes.

[6] Das RK, Banerjee S, Shapiro BH (2013). Extensive sex and/or hormone-dependent expression of rat housekeeping genes. Endocr. Res..

[7] Fan X (2020). High-fat diet alters the expression of reference genes in male mice. Front. Nutr..

[8] Li QQ, Skinner J, Bennett JE (2012). Evaluation of reference genes for real-time quantitative PCR studies in Candida glabrata following azole treatment. BMC Mol. Biol..

[9] Griessl M, Gutknecht M, Cook CH (2017). Determination of suitable reference genes for RT-qPCR analysis of murine Cytomegalovirus in vivo and in vitro. J. Virol. Methods.

[10] Sumbria D, Berber E, Mathayan M, Rouse BT (2020). Virus infections and host metabolism-can we manage the interactions?. Front. Immunol..

[11] Garrison AR, Smith DR, Golden JW (2019). Animal models for Crimean–Congo hemorrhagic fever human disease. Viruses.

[12] Wong KT (2003). A golden hamster model for human acute Nipah virus infection. Am. J. Pathol..

[13] Tang-Huau T-L, Feldmann H, Rosenke K (2019). Animal models for Lassa virus infection. Curr. Opin. Virol..

[14] Garrison, A. R. *et al.* A DNA vaccine for Crimean-Congo hemorrhagic fever protects against disease and death in two lethal mouse models. *PLoS neglected tropical diseases* **11**(9), e0005908 (2017). 10.1371/journal.pntd.0005908 PMC5619839 28922426

[15] Bergeron É (2015). Recovery of recombinant Crimean Congo hemorrhagic fever virus reveals a function for non-structural glycoproteins cleavage by furin. PLoS Pathog..

[16] Chua KB (2000). Nipah virus: A recently emergent deadly paramyxovirus. Science.

[17] Albariño CG (2011). Efficient rescue of recombinant Lassa virus reveals the influence of S segment noncoding regions on virus replication and virulence. J. Virol..

[18] Stelzer G (2016). The GeneCards suite: From gene data mining to disease genome sequence analyses. Curr. Protoc. Bioinform..

[19] Ringwald M (2022). Mouse genome informatics (MGI): Latest news from MGD and GXD. Mamm. Genome.

[20] Untergasser A (2012). Primer3-new capabilities and interfaces. Nucleic Acids Res..

[21] Xie F, Xiao P, Chen D, Xu L, Zhang B (2012). miRDeepFinder: A miRNA analysis tool for deep sequencing of plant small RNAs. Plant Mol. Biol..

[22] Xie F, Wang J, Zhang B (2023). RefFinder: A web-based tool for comprehensively analyzing and identifying reference genes. Funct. Integr. Genom..

[23] Silver N, Best S, Jiang J, Thein SL (2006). Selection of housekeeping genes for gene expression studies in human reticulocytes using real-time PCR. BMC Mol. Biol..

[24] Pfaffl MW, Tichopad A, Prgomet C, Neuvians TP (2004). Determination of stable housekeeping genes, differentially regulated target genes and sample integrity: BestKeeper—Excel-based tool using pair-wise correlations. Biotechnol. Lett..

[25] Andersen CL, Jensen JL, Ørntoft TF (2004). Normalization of real-time quantitative reverse transcription-PCR data: A model-based variance estimation approach to identify genes suited for normalization, applied to bladder and colon cancer data sets. Cancer Res..

[26] Vandesompele J (2002). Accurate normalization of real-time quantitative RT-PCR data by geometric averaging of multiple internal control genes. Genome Biol..

[27] Sievers F (2011). Fast, scalable generation of high-quality protein multiple sequence alignments using Clustal Omega. Mol. Syst. Biol..

[28] Crooks GE, Hon G, Chandonia JM, Brenner SE (2004). WebLogo: A sequence logo generator. Genome Res..

[29] Neerukonda SN, Katneni UK, Golovan S, Parcells MS (2016). Evaluation and validation of reference gene stability during Marek’s disease virus (MDV) infection. J. Virol. Methods.

[30] Fu Y (2020). A novel strategy facilitates reference gene selection by RT-qPCR analysis in kidney yang deficiency syndrome mice infected with the influenza A (H1N1) virus. Biomed. Res. Int..

[31] Zivcec M, Safronetz D, Haddock E, Feldmann H, Ebihara H (2011). Validation of assays to monitor immune responses in the Syrian golden hamster (*Mesocricetus auratus*). J. Immunol. Methods.

[32] Muñoz JJ (2021). Ppia is the most stable housekeeping gene for qRT-PCR normalization in kidneys of three Pkd1-deficient mouse models. Sci. Rep..

[33] Tatsumi K (2008). Reference gene selection for real-time RT-PCR in regenerating mouse livers. Biochem. Biophys. Res. Commun..

[34] Yokoyama T (2018). Identification of reference genes for quantitative PCR analyses in developing mouse gonads. J. Vet. Med. Sci..

[35] Das RK, Banerjee S, Shapiro BH (2013). Extensive sex- and/or hormone-dependent expression of rat housekeeping genes. Endocr. Res..

[36] Jung M (2007). In search of suitable reference genes for gene expression studies of human renal cell carcinoma by real-time PCR. BMC Mol. Biol..

[37] Li Y-L, Ye F, Hu Y, Lu W-G, Xie X (2009). Identification of suitable reference genes for gene expression studies of human serous ovarian cancer by real-time polymerase chain reaction. Anal. Biochem..

