# Supplementary material for: Optimal reference genes for RNA tissue analysis in small animal models of hemorrhagic fever viruses
Source: Sci Rep. 2023 Nov 8;13:19384. doi: 10.1038/s41598-023-45740-w (PMC10632498; doi:10.1038/s41598-023-45740-w)
Supplement: Supplementary file 1 — Supplementary Figures. [file 41598_2023_45740_MOESM1_ESM.docx]

**Optimal reference genes for RNA tissue analysis in small animal models of hemorrhagic fever viruses**

Katherine A. Davies^12^, Stephen R. Welch^1^, Teresa E. Sorvillo^1^, JoAnn D. Coleman-McCray^1^, María Laura Martin^3^, Julia M. Brignone^3^, Joel M. Montgomery^1^, Christina F. Spiropoulou^1^, Jessica R. Spengler^1*^

^1^Viral Special Pathogens Branch, Division of High-Consequence Pathogens and Pathology, Centers for Disease Control and Prevention, Atlanta, GA, USA

^2^U.S. Department of Agriculture, Agricultural Research Service, Zoonotic and Emerging Disease Research Unit, National Bio and Agro-Defense Facility, Manhattan, KS, USA

^3^Departamento Investigación, Instituto Nacional de Enfermedades Virales Humanas (INEVH) “Dr. Julio I. Maiztegui,” Pergamino, Argentina

**Supplementary Figures**

**Figure S1.** **Variation of reference gene expression in tissue RNA isolated from hamsters, mice, and guinea pigs.**

**Figure S2. Heat map of reference gene relative stability scores from mouse, hamster, and guinea pig tissues.**

**Figure S3. Utility of the pan-rodent *Ppia* assay during epidemiological investigations of rodents.**

**
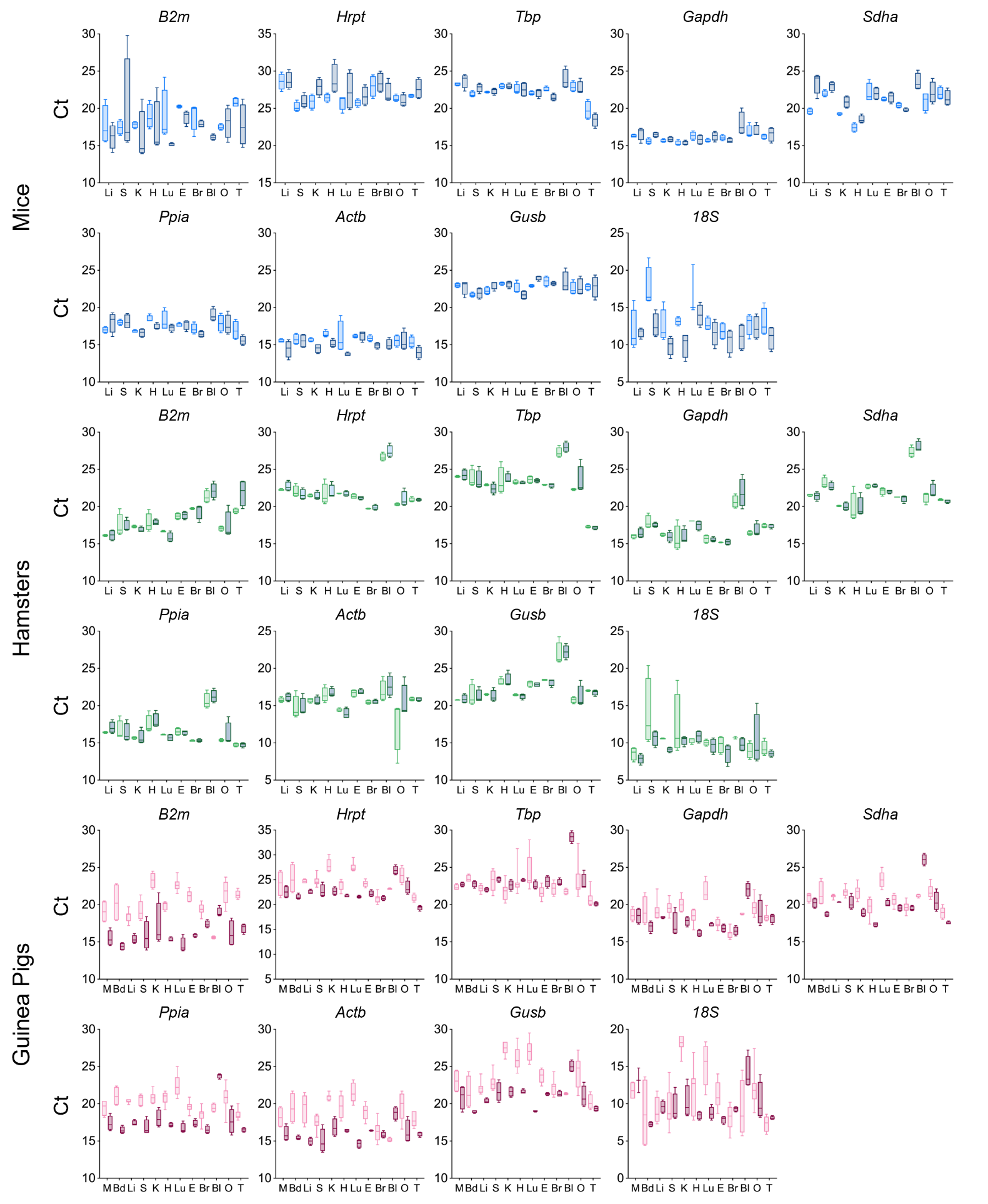
**

**Figure S1.** **Variation of reference gene expression in tissue RNA isolated from hamsters, mice, and guinea pigs.** Expression profiles of 8 reference genes in tissues collected from either naïve animals or animals infected with NiV (hamsters), CCHFV (mice), or LASV (guinea pigs). C_t_ values (n = 3–4) are presented as a box plot, with median values indicated by a line. Minimum and maximum C_t_ values are indicated by error bars. Li, liver; S, spleen; K, kidney; H, heart; Lu, lung; E, eye; Br, brain; Bl, blood; O, ovary; T, testis; M, mammary; Bd, bladder.

**
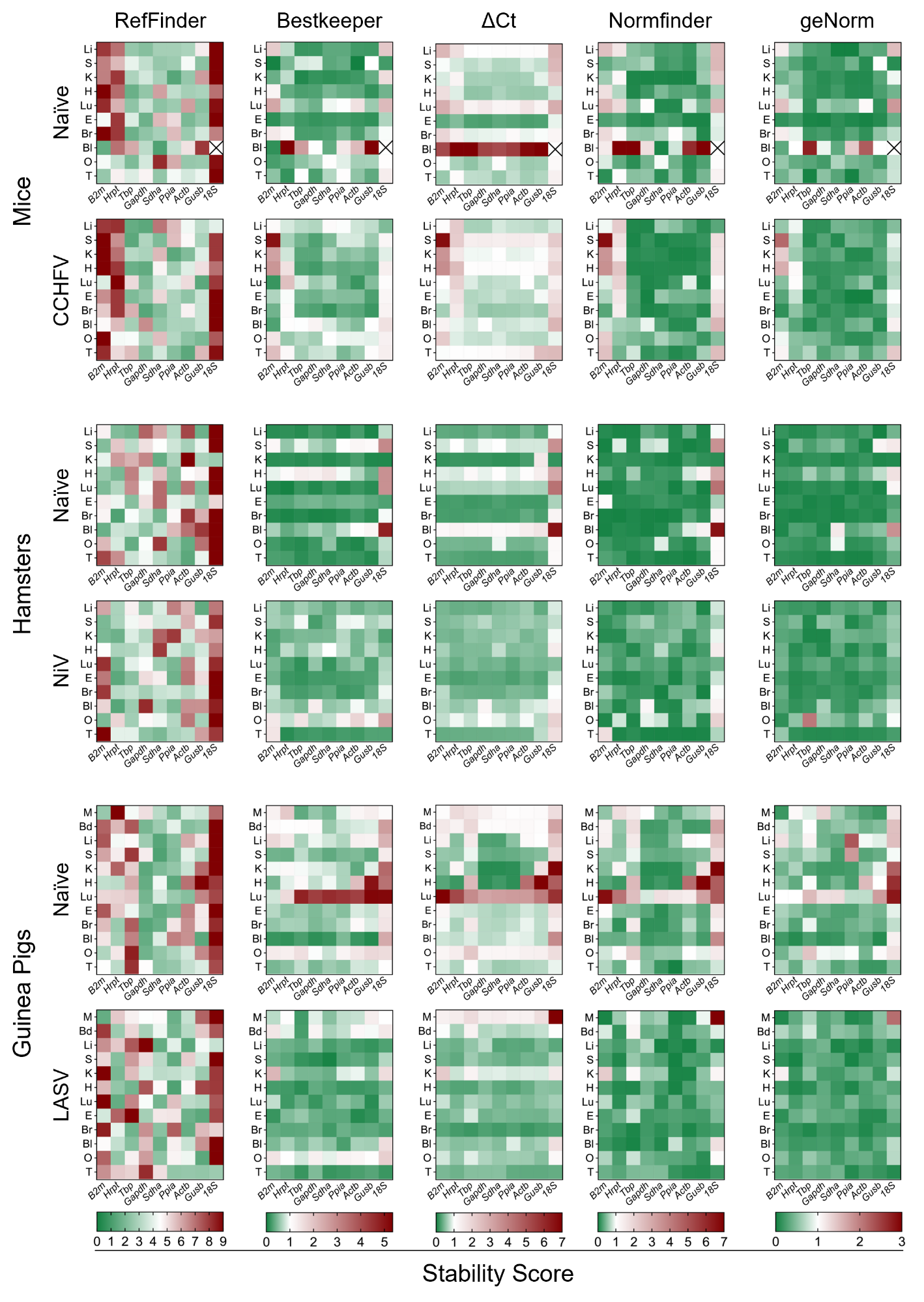
Figure S2. Heat map of reference gene relative stability scores from mouse, hamster, and guinea pig tissues.** Relative gene stability scores as analyzed using RefFinder, Bestkeeper, ∆C_t_ method, Normfinder, or GeNorm are depicted. Each score is determined from 3–4 independent replicates. Red indicates lower stability and green indicates higher stability. Stability scores from naïve animals and virus-infected animals are shown separately. Li, liver; S, spleen; K, kidney; H, heart; Lu, lung; E, eye; Br, brain; Bl, blood; O, ovary; T, testis; M, mammary; Bd, bladder.

**
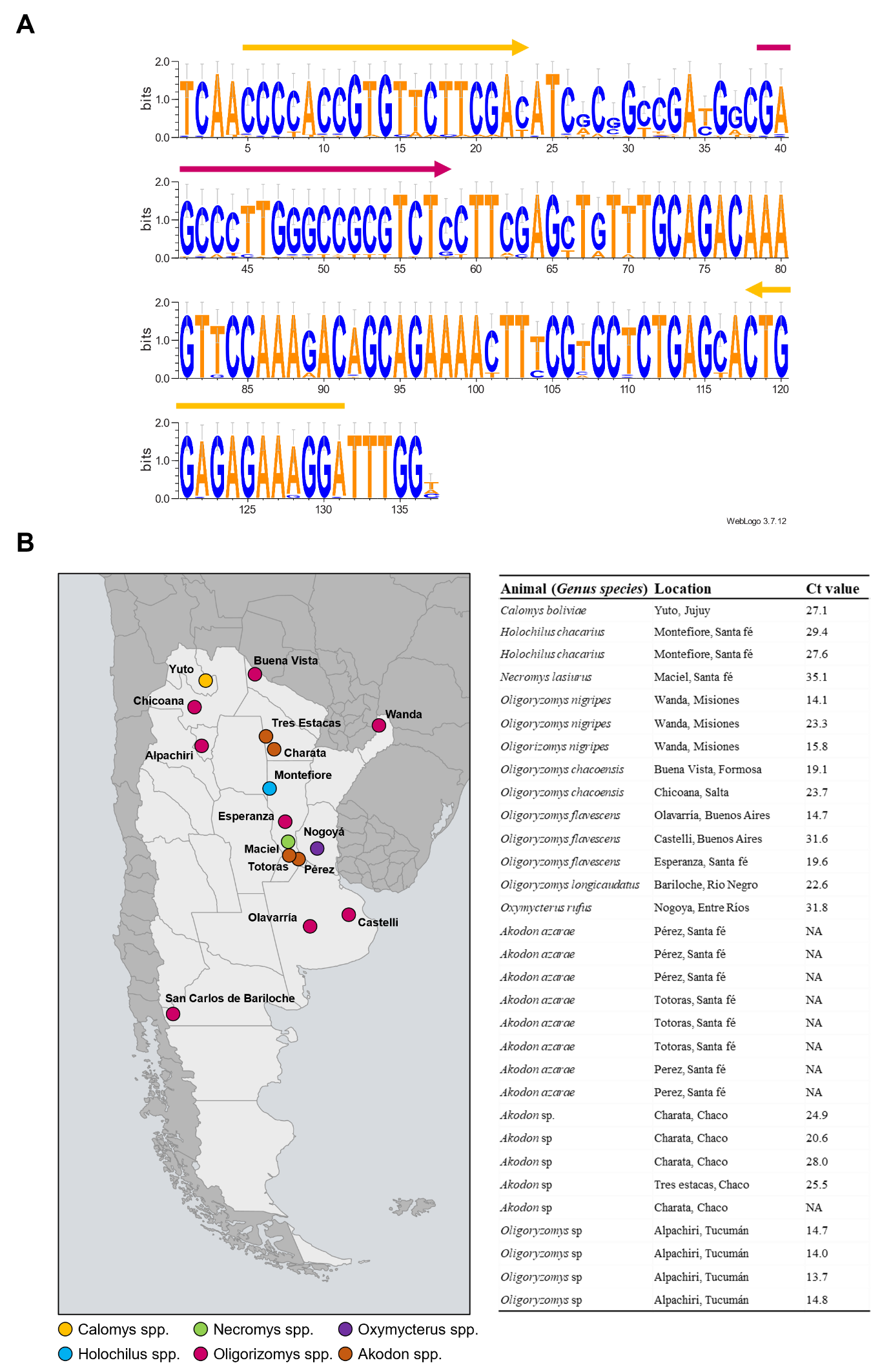
**

**Figure S3.** **Utility of the pan-rodent *Ppia* assay during epidemiological investigations of rodents. (A)** Sequence logo generated from the *Ppia* gene sequence of 26 rodents: *Nannospalax galili* (XM_008834091), *Heterocephalus glaber* (XM_004839898), *Castor canadensis* (XM_020163974), *Cricetulus griseus* (XM_007645276), *Rattus norvegicus* (NM_017101), *Mus musculus* (NM_008907), *Marmota flaviventris* (XM_027938340), *Mesocricetus auratus* (XM_005086775), *Dipodomys ordii* (XM_013036860), *Mus pahari* (XM_021210601), *Peromyscus maniculatus bairdii* (XM_006973006), *Jaculus jaculus* (XM_004652917), *Meriones unguiculatus* (XM_021632741), *Grammomys surdaster* (XM_028777605), *Arvicanthis niloticus* (XM_034509228), *Onychomys torridus* (XM_036201508), *Arvicola amphibius* (XM_038330452), *Microtus oregoni* (XM_041669255), *Microtus ochrogaster* (XM_013353354), *Cavia porcellus* (XM_003465805), *Chinchilla lanigera* (XM_005396653), *Octodon degus* (XM_004630360), *Fukomys damarensis* (XM_010624925), *Ictidomys tridecemlineatus* (XM_005319249), *Urocitellus parryii* (XM_026399465), and *Mastomys coucha* (XM_031340267). Sequences have been truncated at the locations of the pan-rodent *Ppia* primer sites. Primer binding sites are indicated in yellow (forward and reverse), and probe is indicated in pink. **(B)** Map of Argentina (created using mapchart.net 2023) indicating locations at which each genus of rodent was collected. All rodents with positive or negative results in the pan-rodent *Ppia* assay are listed to either genus or species level, along with the location of trapping.
